# A Comparative Study of Analgesic Efficacy of Epidural Ropivacaine With Dexmedetomidine Versus Ropivacaine With Ketamine in Adult Patients Undergoing Elective Lower Limb Surgery

**DOI:** 10.7759/cureus.26792

**Published:** 2022-07-12

**Authors:** Shalini Gujral, Bhupendra Singh, Rajendra K Solanki, Babita Babita, Seema Yadav, Rajendra K Pipal, Dharmendra K Pipal, Vibha Rani Pipal

**Affiliations:** 1 Anaesthesia, Dr Sampurnanand Medical College, Jodhpur, IND; 2 Anaesthesia, All India Institute of Medical Sciences, Raebareli, IND; 3 Anaesthesia, Divisional Railway Hospital, Jodhpur, IND; 4 Anaesthesia, Jaipur National University (JNU) Medical College and Hospital, Jaipur, IND; 5 Orthopaedics, Geetanjali Medical College, Udaipur, IND; 6 General, Colorectal, and Minimal Access Surgery, All India Institute of Medical Sciences, Gorakhpur, IND; 7 Obstetrics and Gynaecology, All India Institute of Medical Sciences, Gorakhpur, IND

**Keywords:** lower limb surgery, ketamine, dexmedetomidine, ropivacaine, epidural

## Abstract

Introduction: Adjuvating of the epidural block with local anaesthetics during lower limb surgeries improves Intraoperative as well as postoperative analgesia. A comparison of epidural ropivacaine plus dexmedetomidine (RD) versus ropivacaine plus ketamine (RK) was done in terms of quality of the motor and sensory blockade, changes in hemodynamic parameters, and efficacy of analgesia.

Methods: A prospective randomized parallel double-blind study was conducted on 68 patients of the American Society of Anaesthesiologists (ASA) grade 1 and 2, ages 18 to 75 years, which were divided into two groups (RD and RK; 34 patients in each group). After receiving a loading dose through an epidural catheter consisting of 20ml of 0.5% ropivacaine, the epidural infusion was started after an hour of surgery at 5ml/hrs of 0.2% ropivacaine with 1µg/ml dexmedetomidine in Group RD and at 5ml/hrs of 0.2% ropivacaine with 0.5mg/ml ketamine in Group RK for 48 hours. Both groups were compared regarding the onset of sensory and motor block, resolution of sensory and motor block, hemodynamic parameters, analgesic efficacy, and total rescue analgesic requirement in 48 hours.

Results: A significant difference was observed in the time of resolution of sensory blockade which was 9.77±2.38 hrs in the RD group as compared to 7.79±1.82 hrs in the RK group (p-value 0.0003) and the time of resolution of motor block was 6.53±2.44 hrs in the RD group compared to 4.58±0.83 hrs in the RK group (p-value 0.001).

Conclusions: Epidural dexmedetomidine significantly increases the duration of analgesia and duration of the motor blockade in comparison to ketamine.

## Introduction

The current International Association for the Study of Pain (IASP) definition of pain as "pain an unpleasant sensory and emotional experience associated with, or resembling that associated with, actual or potential tissue damage” [1]. It affects the physiology of almost all other systems including respiratory, cardiovascular, and metabolic profiles, and thus it increases morbidity [2].

Michael et al. demonstrated that neuraxial block lowers the incidence of venous and pulmonary embolism, as well as the need for blood transfusions and respiratory compromise following upper abdominal surgeries [3]. It has also been shown that the body's stress response decreases, which may have beneficial effects on the cardiovascular system, such as a reduction in perioperative ischemia. Other advantages are no poly-pharmacy, airway manipulations, and ideal operative conditions [4]. Postoperative analgesia is an essential component of patient care and it plays a critical role in facilitating early recovery and reducing morbidity [5]. In 40% of cases, severe pain could lead to a hyperalgesia syndrome called persistent postoperative pain (PPP). This should be treated as quickly and aggressively as possible to avoid a delay in discharge from the hospital. Furthermore, 18.3% of patients report that the severity of their pain is moderate to severe [6]. Because of this, it is in the best interest of anesthesiologists to know how serious this problem is as well as all of the drugs that are used to prevent and treat pain after surgery. Even though opioids like fentanyl and morphine are often used to treat pain but the pain isn't always completely relieved, subsequently patients often develop a tolerance to the drugs, which makes them more likely to become addicted [7]. Epidural analgesia expedites the return of normal gastrointestinal motility and decreases the amount of opioid medication required [8]. Epidural local anaesthetics with adjuvants such as clonidine, dexmedetomidine, opioids, ketamine [9,10], and midazolam improve both the duration and quality of pain relief. Additionally, It also decreases the dose of local anaesthetic agents which leads to lessening the possibility of systemic and local anaesthetic toxicity as well as the degree of motor block [11].

There are only a few studies that compare epidural ropivacaine with dexmedetomidine and ropivacaine with preservative-free (PF) ketamine for lower limb surgery. So, our study aimed to evaluate the efficacy of postoperative analgesia, sensory and motor blockade, hemodynamic stability and requirement of rescue analgesia in lower limb surgeries. 

## Materials and methods

The study was carried out in the Department of Anaesthesiology in association with the Department of Orthopaedics affiliated with Dr Sampurnanad Medical College, Jodhpur. The institutional ethics committee approval no. was F.1/Acad/MC/JU/18/14024 and the CTRI trial no. is REF/2019/09/028066.

The present study included patients with ASA grades 1 and 2, ages 18 to 75 years, a height of 145 to 180 cm, a weight of 50 to 85 kg, a body mass index (BMI) of 20 to 30, and of either gender. Those having an injection site infection, coagulation or bleeding disorder, severe hypervolemia, aortic or mitral stenosis, right to left shunting, increased intracranial pressure, pulmonary hypertension, history of allergy to local anaesthetics, pregnant and lactating females, anatomical deformities of the spine, conduction abnormalities, and heart rate (HR) <60 beats/min were excluded from the study.

Sample size estimation

In a pilot study done by Sonawane et al. [12], the duration of analgesia in group ropivacaine with dexmedetomidine (RD) was 350.65±58.25 min and group ropivacaine with ketamine (RK) was 311.14±51.45 min. On the basis of this pilot study keeping power of 80% and with a confidence interval of 95% (α= 0.05), the calculated sample size came out to 34 patients for each group in our study.

An in-depth history was taken, along with a comprehensive general physical examination that included an airway assessment with spine and systemic examination. All patients had gone through the standard pre-anaesthesia check-up. In OT, the patient was identified, and consent and fasting status were checked. IV line secured using 18G cannula and each subject has received preload Ringer Lactate 15ml/kg. All standard monitoring was attached and baseline parameters like HR, blood pressure (BP), mean arterial pressure (MAP), and oxygen saturation (SpO2) were noted. Under all aseptic precautions, L3-4 or L2-3 interspaces were identified by palpation and the overlying skin was infiltrated with 2% lignocaine. After infiltration, an 18 G Tuohy needle was advanced by a resident under the supervision of a consultant. The epidural space was identified by the loss of resistance (LOR) technique with normal saline and the catheter was inserted 4 to 5 cm in the epidural space. The catheter’s position was tested with 3ml of lignocaine 1.5% with 1:200000 adrenaline preparations to exclude intrathecal and intravascular placement. Group allocation was done by opaque, sealed envelopes, and randomization was done by computer-generated numbers. The study was double-blind so the patient and investigator were not aware of the group and given intervention. An initial loading epidural dose of 0.5% ropivacaine (20 ml) was administered to each patient. Surgery started after the sensory blockade was achieved at the dermatome T10 level. According to the protocol for the assigned study group, the epidural infusion was started one hour after the incision.

Group RD patients received ropivacaine 0.2% + dexmedetomidine 1µg/ml infusion at 5ml/hr epidurally for 48 hrs (total volume of 240 ml). Group RK patients received ropivacaine 0.2% + ketamine 0.5mg/ml infusion at 5ml/hr epidurally for 48 hrs (total volume of 240 ml).

Parameters studied

The patient's parameters such as HR, systolic blood pressure (SBP), diastolic blood pressure (DBP), mean blood pressure (MBP), SpO2, and Visual Analogue Score (VAS) were monitored every five minutes for the first 30 minutes, every hour until the fourth hour, every two hours until the sixth hour, and every six hours until the 48th hour. Thereafter, patient was monitored in the ward as per institute protocol.

The onset and the time of resolution of motor and sensory block, hemodynamic stability, total rescue analgesic requirement in 48 hours, and any side effects were studied. The time interval between the administration of epidural block and sensory blockage up to the T10 level, measured using the pinprick method with a 22G hypodermic needle in the midline, was considered the time of onset of sensory blockade. The time of onset of motor blockade was defined as the interval between administrations of epidural block till the time when the patient achieved modified Bromage Grade 3. Degree of motor blockade was assessed with the modified Bromage scale by Breen (in non-operated limb) [13]. The time of resolution for the sensory blockade was defined as the time of regression from T10 level to L5 level as assessed by the pinprick method.

Time for resolution of motor blockade was defined as the time of regression from modified Bromage Grade 3 to 6. The degree of pain relief was assessed using the VAS system, with 0 representing no pain at all and 10 representing the worst imaginable pain ever; if at any stage the VAS score was more than 4, rescue analgesia was given with intravenous diclofenac 150 mg and the total requirement of rescue analgesia was noted till 48 hours.

Hypotension, hypertension, bradycardia, tachycardia, shivering, nausea, vomiting, and respiratory depression were all observed and treated as warranted. Bradycardia was defined as a 20% reduction in heart rate from baseline. Tachycardia was defined as a 20% rise in heart rate over baseline. Hypertension was defined as a 20% increase in MBP over baseline, whereas hypotension was defined as a 20% reduction in MBP over baseline. If the respiration rate was less than 10 per minute at any point during the study, it was considered respiratory depression. The epidural catheter was removed two days after the surgery. All patients were observed postoperatively for any procedure-related complications like post-dural puncture headache, backache, migration of catheter, transient neurological symptoms, etc.

Consort flow chart

**Figure 1:**
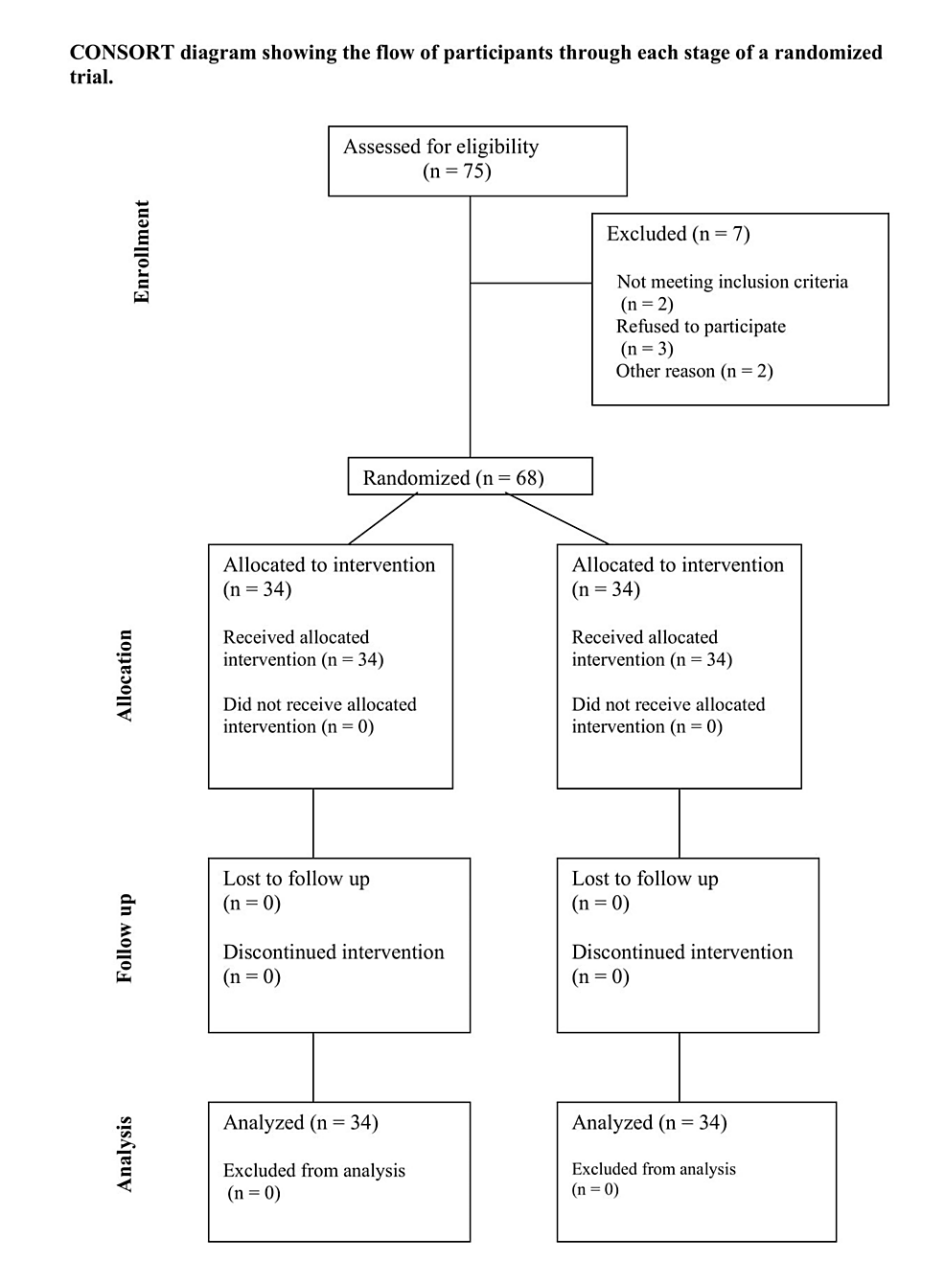
Consort Flow Chart

A total of 75 patients were enrolled in the study. Out of them, seven were excluded (Figure 1) due to various reasons mentioned in the consort chart. Therefore, a total of 68 patients were allocated and divided into two groups of 34 patients each.

Statistical analysis

All statistical analyses were performed by using SPSS 22.0 software package (IBM Corp., Armonk, NY, USA). Yates continuity correction test *(Chi-square test), Fisher’s exact test and 25 Fisher---Freeman---Halton test, Unpaired t-test will be used for comparison of qualitative data. All data will be summarized as mean ± SD for continuous variables, numbers, and percentages for categorical variables. A p < 0.05 was accepted as statistically significant.

## Results

The demographic data i.e age, height, weight, ASA grade, M: F ratio and duration of surgery and level of catheter fixation are comparable in both groups and statistically nonsignificant (Table 1).

**Table 1:** Demographic data ASA: American Society of Anaesthesiologists

Parameter			RD Group	RK Group	P value
Age in yrs			59.50±12.77	57.74±15.67	.612
Weight in kgs			71.18±10.07	70.29±12.38	.748
Height in cms			166.91±8.65	165.55±10.61	.566
ASA Grade	I		17	17	1.00
	II		17	17	1.00
BMI (Body mass index)			25.43±2.31	25.46±2.40	.961
Gender		Male	17	17	
		Female	17	17	
Duration of surgery hrs			2.88±0.37	2.86±0.38	.81
Level of catheter fixation in cm			10.73±1.26	10.5±0.66	.339

**Figure 2:**
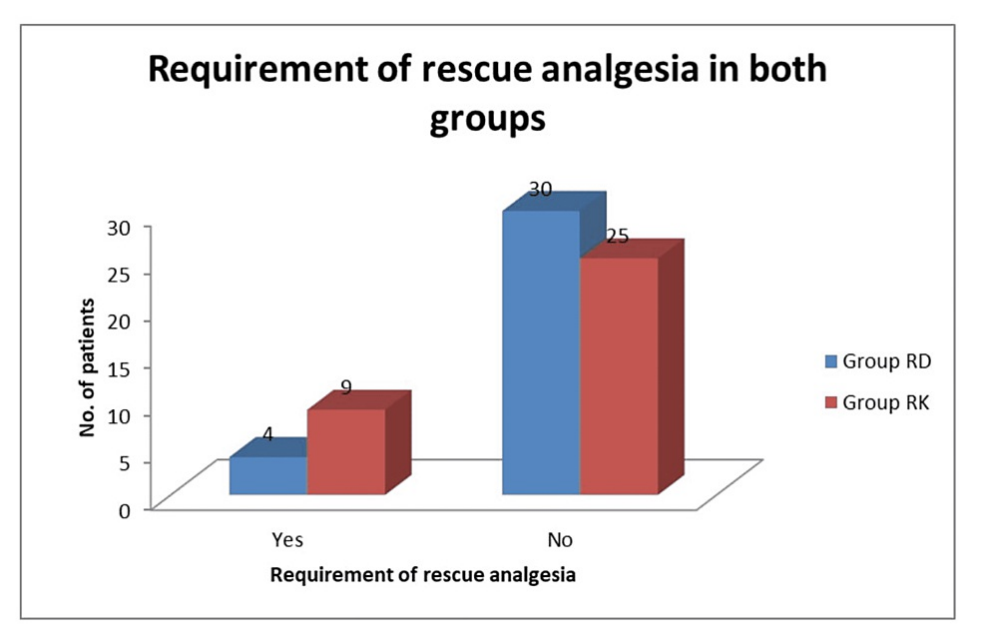
Requirement of rescue analgesia in both the groups RD: ropivacaine plus dexmedetomidine, RK: ropivacaine plus ketamine

Time of onset of motor block, sensory block and total dose of rescue analgesia are comparable in both groups and statistically nonsignificant (Table 2, Figure 2).

**Table 2:** Time of onset of sensory and motor block in both the groups RD: ropivacaine plus dexmedetomidine, RK: ropivacaine plus ketamine

Parameter	RD Group	RK Group	P value
Time of onset of motor block (Bromage 3) in min	11.03±3.66	9.82±2.29	0.107
Time of onset of sensory block (T10 level) in min	8.79±4.07	7.62±2.16	0.141
Time of resolution of motor blockade (3 grade to grade 6) in hrs	6.53±2.45	4.59±0.84	0.0001
Time of resolution of sensory block to L5 in hrs	9.78±2.39	7.79±1.82	0.0003
Total required rescue analgesia	26.47±78.07	44.11±78.59	0.356

**Figure 3:**
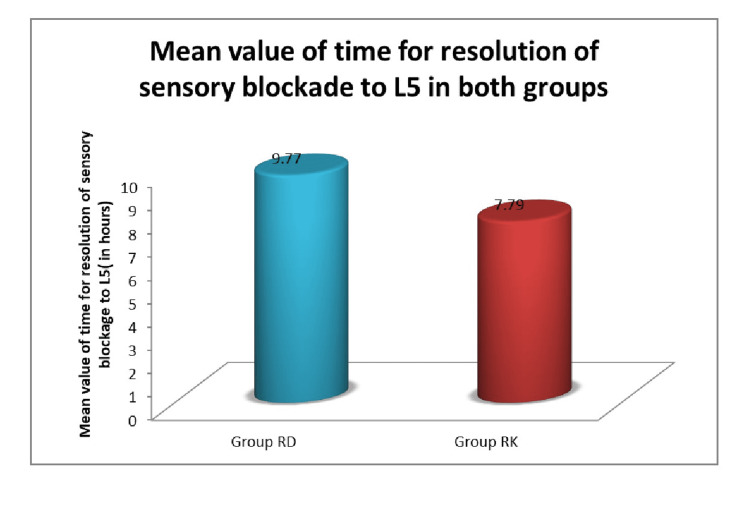
Mean value of time for resolution of sensory bolckade to L5 in both groups RD: ropivacaine plus dexmedetomidine, RK: ropivacaine plus ketamine

**Figure 4:**
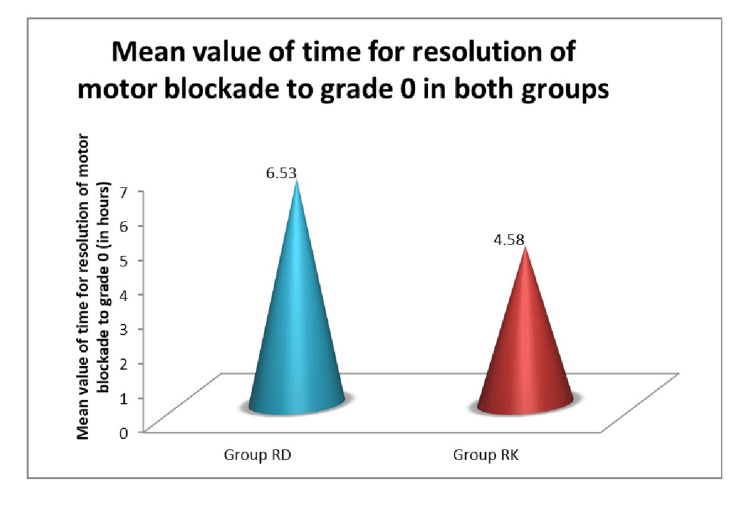
Mean value of time for resolution of motor blockade in both groups RD: ropivacaine plus dexmedetomidine, RK: ropivacaine plus ketamine

The mean value of time for resolution of motor blockade was greater in Group RD (6.53±2.44 hrs) as compared to Group RK (4.58±0.83 hrs) and the P-value was ˂ 0.0001. The mean value of time for resolution of the sensory blockade to L5 was more in Group RD (9.77±2.38 hrs) as compared to Group RK (7.79±1.83 hrs) and the P-value was 0.0003 (Figures 3, 4).

**Figure 5:**
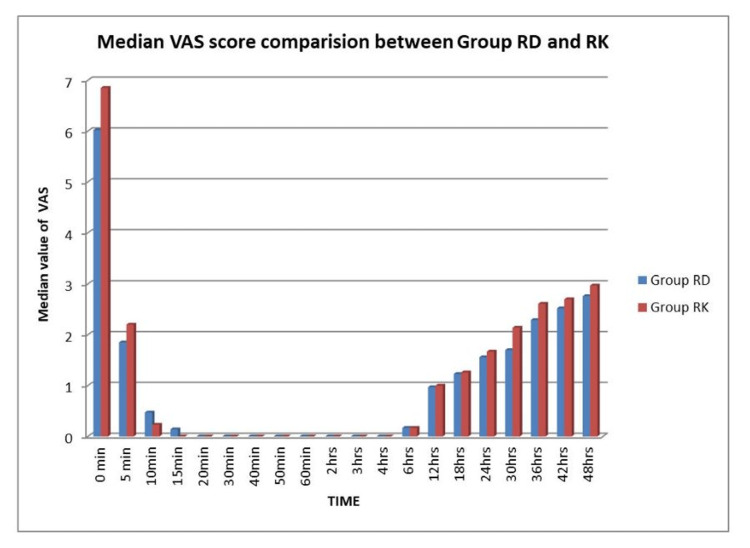
Median Visual Analogue Scores (VAS) Score comparison between the two groups RD: ropivacaine plus dexmedetomidine, RK: ropivacaine plus ketamine

The median VAS scores of groups RD and RK were analyzed for up to 48 hours using an unpaired t-test. The VAS score was significant at 0 min, 30 hours, and 48 hours (P-value 0.013, 0.013, and 0.025, respectively) (Table 3, Figure 5).

**Table 3:** Visual Analogue Scores (VAS) in both groups RD: ropivacaine plus dexmedetomidine, RK: ropivacaine plus ketamine

Time	VAS				t value	p value
	Group RD		Group Rk			
	Median		Median	Mean±SD		
0 min	6	6.02±1.35	7	6.85±1.30	2.548	0.013
5 min	2	1.85±1.20	2	2.20±0.84	1.638	0.106
10min	0	0.47±0.70	0	0.23±0.49	1.589	0.116
15min	0	0.14±0.43	0	0.00±0.00	NA	NA
20min	0	0.00±0.00	0	0.00±0.00	NA	NA
30min	0	0.00±0.00	0	0.00±0.00	NA	NA
40min	0	0.00±0.00	0	0.00±0.00	NA	NA
50min	0	0.00±0.00	0	0.00±0.00	NA	NA
60min	0	0.00±0.00	0	0.00±0.00	NA	NA
2hrs	0	0.00±0.00	0	0.00±0.00	NA	NA
3hrs	0	0.00±0.00	0	0.00±0.00	NA	NA
4hrs	0	0.00±0.00	0	0.00±0.00	NA	NA
6hrs	0	0.17±0.45	0	0.17±0.38	0	0.999
12hrs	1	0.97±0.45	1	1±0.00	NA	NA
18hrs	1	1.23±0.60	1	1.26±0.61	0.198	0.843
24hrs	1	4.55±0.78	2	1.67±0.63	0.677	0.5
30hrs	2	1.79±0.59	2	2.14±0.55	2.531	0.013
36hrs	2	2.29±0.57	2	2.61±0.92	1.733	0.087
42hrs	3	2.52±0.50	3	2.70±0.52	1.412	0.162
48hrs	3	2.76±0.43	3	2.97±0.30	2.288	0.025

In some patients, side effects like hypertension and tachycardia were associated with the RK group and bradycardia, hypotension, nausea and vomiting were associated with the RD group. None of patients in either group reported respiratory depression and shivering (Table 4).

**Table 4:** Adverse effect in both groups (p value 0.082) RD: ropivacaine plus dexmedetomidine, RK: ropivacaine plus ketamine

Adverse effects	Group RD		Group RK	
	N	%	N	%
Bradycardia	8	23.53	0	0.00
Hypotension	7	20.59	0	0.00
Hypertension	0	0.00	4	11.76
Tachycardia	0	0.00	6	17.65
Nausea and vomiting	2	5.88	0	0.00
Shivering	0	0	0	0
Respiratory depression	00	0	0	0
Total	17	50.00	10	29.41

## Discussion

Epidural anaesthesia is the most frequently employed approach for perioperative analgesia in both lower limb and lower abdominal procedures [14]. Ropivacaine appears to be an important alternative due to its efficacy. It also has a lower probability of motor block as well as CNS and cardiac toxicity [15]. Sensory and motor blocks induced by Ropivacaine depend on the dose as well as the age of the patient [16]. Dexmedetomidine has been studied extensively and is a highly selective α2 receptor agonist that has been used clinically to generate the desired effects in regional anaesthesia [17].

Peripheral and central sensitization of nociceptors can be prevented by an N-methyl-D-aspartate antagonist (NMDA) antagonist like ketamine. The addition of 0.4 mg/ml of ketamine to a multimodal patient-controlled epidural analgesia (PCEA) regimen improved post-operative pain relief and reduced analgesic consumption, according to Chia et al. [18]. Postoperative analgesia was studied by Naguib et al. [19], who found that 30 mg of epidural ketamine was a safe and effective approach. Respiratory depression, mental disturbances, cardiac toxicity, bladder irritability and dysfunction, or any other neurologic deficits were not observed in any of the patients treated.

In this study, we compared how much heart rate and blood pressure including systolic, diastolic, mean arterial, and SpO2 changed from their baseline value to 48 hours of epidural catheterization at different time intervals and were comparable between groups. The time to reach sensory level T10 in Group RD (8.79±4.06 minutes) was found to be delayed compared to Group RK (7.61±2.16 minutes). The difference (p = 0.141) was statistically insignificant. In a study conducted by Arunkumar et al. [20], they observed similar results regarding the onset of sensory blockade using a cold swab at T10 in the patients receiving dexmedetomidine (8.53±1.81 min).

Group RD experienced motor blockade on average after 11.02±3.65 minutes, while Group RK experienced it after 9.82±2.28 minutes, which was not statistically significant (p-value 0.107). The onset time to complete motor blockade using the modified Bromage scale with ropivacaine + dexmedetomidine was 18.16±4.52 minutes in a study done by Shaikh et al. [21]. The time of onset of motor blockade was the interval between administration of epidural block to achieve Bromage grade 3, therefore, the time of motor onset was shorter in our study.

Resolution of sensory block to the L5 dermatome was earlier in Group RK (7.79±1.82 hours) when compared to Group RD (9.77±2.38 hours). The difference between the two groups was statistically significant, having a P-value of 0.003. Sonawane et al. conducted a similar study and discovered that the receding time for the sensory blockade was significantly longer in Group RD (9.33±4.34 hours) than in Group RK (7.03±3.79 hours) and p-value was 0.033. In a study conducted by Arunkumar et al., they found that the duration of sensory analgesia was 316±31.15 min, i.e., 5.27±0.52 hrs in group RD, which was much shorter than in our study. This could be because they only gave the bolus of dexmedetomidine once, whereas in our study we used a continuous infusion.

Our study has shown a significantly prolonged time for resolution of the motor block from Bromage grade 3 to 6 in Group RD (6.53±2.44 hours) as compared to Group RK (4.58±0.83 hours). The difference between the two groups was statistically significant, having a P-value < 0.0001. Sonawane et al. also had similar results regarding the receding time of the motor block, which was 7.10±3.53 hours in Group RD and 3.80±1.49 hours in Group RK (P-value 0.000).

The pain was assessed using the VAS score in our study. We found that the VAS score was mostly statistically non-significant between both groups except at 0 min, 30 hours, and 48 hours. The number of patients requiring rescue analgesia in Group RK was higher (nine patients) as compared to Group RD (four patients), but the difference was statistically insignificant. Also, the mean value of total rescue analgesia required in Group RK (44.11±78.59) was higher than in Group RD (26.47±78.07) but the difference was statistically not significant (P-value 0.356). These results were similar to the study conducted by Sonawane et al., Rabin et al. and Pandya et al. [12,22,23]. 

The current study's limitations included a smaller sample size and an evaluation that lasted only 48 hours postoperatively. Furthermore, it was only done for lower limb surgery. A larger sample size study, as well as evaluation in other surgeries, such as abdominal and thoracic, are required.

## Conclusions

Epidural dexmedetomidine significantly prolongs the duration of motor and sensory blocks as compared to ketamine. However, relatively a greater number of patients required rescue analgesia in the ketamine group, but this was statistically insignificant. Side effects such as hallucination and delirium were not observed in any patient. So, it is difficult to convincingly prove which drug is superior to another in this relatively small sample size. So, we would suggest that each centre should select techniques and drugs for post-operative analgesia that are suitable depending upon the availability of required equipment and drugs, cost-effectiveness, ease of the procedure, and patient satisfaction.
